# Nitrogen-Doped Diamond-like Coatings for Long-Term Enhanced Cell Adhesion on Electrospun Poly(ε-caprolactone) Scaffold Surfaces

**DOI:** 10.3390/polym16243524

**Published:** 2024-12-18

**Authors:** Semen Goreninskii, Yuri Yuriev, Artem Runts, Elisaveta Prosetskaya, Evgeniy Melnik, Tuan-Hoang Tran, Elizaveta Sviridova, Alexey Golovkin, Alexander Mishanin, Evgeny Bolbasov

**Affiliations:** 1 Additive Technologies Center, Tomsk Polytechnic University, Tomsk 634050, Russia; sig1@tpu.ru; 2 B.P. Veinberg Research and Educational Centre, Tomsk Polytechnic University, Tomsk 634050, Russia; yurjev@tpu.ru; 3 Microwave Photonics Lab, V.E. Zuev Institute of Atmospheric Optics SB RAS, Tomsk 634055, Russia; aar74@tpu.ru (A.R.); eap47@tpu.ru (E.P.); 4 Research School of Chemistry & Applied Biomedical Sciences, Tomsk Polytechnic University, Tomsk 634050, Russia; eymelnik@tpu.ru (E.M.); cungbinh9327@gmail.com (T.-H.T.); evs31@tpu.ru (E.S.); 5 Almazov National Medical Research Center, St. Petersburg 197341, Russia; golovkin_a@mail.ru (A.G.); mishaninssma@yandex.ru (A.M.)

**Keywords:** poly(ε-caprolactone) scaffold, diamond-like coatings, plasma modification, stem cells, biocompatibility

## Abstract

Electrospun poly(ε-caprolactone) (PCL)-based scaffolds are widely used in tissue engineering. However, low cell adhesion remains the key drawback of PCL scaffolds. It is well known that nitrogen-doped diamond-like carbon (N-DLC) coatings deposited on the surface of various implants are able to enhance their biocompatibility and functional properties. Herein, we report the utilization of the pulsed vacuum arc deposition (PVAD) technique for the fabrication of thin N-DLC coatings on the surface of electrospun PCL scaffolds. The effect of N-DLC coating deposition under various nitrogen pressures on the morphological, mechanical, physico-chemical, and biological properties of PCL scaffolds was investigated. It was established that an increase in nitrogen pressure in the range from 5 × 10^−3^ to 5 × 10^−1^ Pa results in up to a 10-fold increase in the nitrogen content and a 2-fold increase in the roughness of the PCL fiber surface. These factors provided the conditions for the enhanced adhesion and proliferation of human mesenchymal stem cells (MMSCs) on the surface of the modified PCL scaffolds. Importantly, the preservation of N-DLC coating properties determines the shelf life of a coated medical device. The elemental composition, tensile strength, and surface human MMSC adhesion were studied immediately after fabrication and after 6 months of storage under normal conditions. The enhanced MMSC adhesion was preserved after 6 months of storage of the modified PCL-based scaffolds under normal conditions.

## 1. Introduction

Tissue engineering combines the approaches of biomedical engineering, medicine, chemistry, physics, and material science to restore damaged organs and tissues. Tissue engineering scaffolds are complex structures that serve to facilitate the growth and proliferation of various cells [1]. Due to their high biocompatibility, elasticity, and surface area, electrospun poly(ε-caprolactone) (PCL)-based scaffolds are suggested and applied for the restoration of skin, bone, vascular, and other tissues and organs. However, limited cell adhesion to the surface of PCL-based electrospun scaffolds due to the low functional activity of PCL results in poor tissue integration [2]. Thus, the surface functionalization of electrospun PCL-based scaffolds is needed for tissue engineering applications.

Several physical and chemical approaches to surface functionalization were suggested to enhance the interaction of cells and tissues with PCL scaffolds [3]. The chemical approaches are mainly based on the treatment of scaffolds with alkaline solutions or organic solvents [4]. These methods necessitate the use of high-purity reagents, strict control of the process parameters, and intensive washing, which may be challenging due to the high surface area and absorptive capacity of the scaffold.

The physical approaches often utilize plasma treatment [5]. However, the low melting point of PCL (~60 °C) limits both the discharge energy and exposure time, as a substantial increase in either would result in the destruction and loss of scaffold functionality [6]. In addition, plasma treatment provides only temporary surface hydrophilization (~1 month) [7,8], limiting the shelf life of the modified PCL scaffolds and resulting in specific storage requirements that complicate clinical applications and the manufacturing process.

The use of the pulsed vacuum arc deposition (PVAD) technique for depositing thin diamond-like carbon (DLC) coatings is looking promising for the effective functionalization of electrospun scaffolds. This method provides a high rate of condensate formation (up to 1 × 10^4^ Å/s) [9] and does not require an acceleration potential to be applied to the substrate. With that, ion energy in the generated carbon plasma is in the range of 40–90 eV, and the substrate temperature does not exceed 70 °C, which makes it possible to deposit thin biocompatible coatings on the surface of electrospun polymer fibers. Additionally, compared to other methods for DLC coating formation, the PVAD technique provides a high rate and area of the coating growth [10].

The PVAD technique allows for coating deposition under an atmosphere of various gases with a wide pressure range, enabling the incorporation of various elements in coatings in order to achieve the required mechanical or biological properties [11]. Liao et al. studied the biocompatibility of N-DLC coatings deposited on silicon substrates [12]. The authors demonstrated that fibroblasts cultured with the N-DLC-coated samples had better viability compared to cells cultured with the platinum-, silicon-, and DLC-coated samples. Numerous groups have demonstrated the possibility of N-DLC coating deposition on polymer substrates. Kyzioł et al. reported the deposition of N-DLC coatings on polyurethane films using the plasma-enhanced chemical vapor deposition method [13]. The fabricated coatings had a high biocompatibility towards osteoblast-like MG-63 cells and keratinocytes. Srinivasan et al. reported the possibility of N-DLC coating deposition on polytetrafluoroethylene films using the ion beam deposition technique for enhanced hemocompatibility [14].

While N-DLC coatings are promising for the functionalization of PCL scaffolds, there are only a few reports in this area. It limits both the application of electrospun PCL scaffolds in tissue engineering and the utilization of N-DLC coatings for the functionalization of complex structures with low melting points (e.g., electrospun PCL scaffolds). It is known that PCL scaffolds can preserve their physico-chemical properties for 6 months after fabrication [15]; however, there are no reports evaluating the biocompatibility of N-DLC-coated electrospun PCL scaffolds after their storage at normal conditions. Such studies are vital, as they not only allow the determining of the shelf life of the device but are also of fundamental interest for biomedical materials science. Therefore, the aim of the present study was to investigate the key structural, physico-chemical, and biological properties of N-DLC-coated PCL scaffolds immediately after modification and after 6 months of storage.

## 2. Materials and Methods

### 2.1. Fabrication of PCL Scaffolds

The scaffolds were fabricated from a 6 wt. % solution of PCL (80,000 g/mol, Sigma, St. Louis, MO, USA) in chloroform (Ecros, Moscow, Russia) using a NANON-01A (MECC Co., Ltd., Fukudo, Ogori-shi, Fukuoka, Japan) electrospinning setup equipped with a steel cylindrical collector (200 mm length, 100 mm diameter) and a 24G needle for the polymer solution supply. All the scaffolds were spun at 20 kV, a 6 mL/h polymer solution feed rate, 50 rpm collector rotation rate, and a needle tip to collector distance of 190 mm. The scaffolds were fabricated at a temperature of 26 °C and a humidity of 23%. After their fabrication, the scaffolds were placed in a VD 115 (Binder, Tuttlingen, Germany) vacuum chamber at a temperature of 40 °C and a pressure of 0.1 Pa for 24 h in order to remove the residual solvents.

### 2.2. Deposition of N-DLC Coatings

The coatings were deposited on the surface of scaffold samples with a size of 50 × 50 mm (thickness of 273 ± 25 µm) via the evaporation of a high-purity graphite cathode (99.99% purity) in a nitrogen (99.99% purity) atmosphere using the setup and regimes described earlier [16]. The following technological parameters were applied for the coating deposition: a 400 V striking voltage, 170 V capacitor voltage, 1 Hz pulse frequency, and 3000 pulses per sample. The pulsed arc was powered by using a 2000 μF capacitor bank. The nitrogen pressure during the deposition process was set at 5 × 10^−3^, 5 × 10^−2^, and 5 × 10^−1^ Pa. The non-coated samples were used as a control group. For the investigation of the elemental composition and biocompatibility changes after storage, the samples were placed in Steripack (PMS, Mersin, Turkey) sterilization bags and stored in a dark, dry place at a temperature of 26 °C and a humidity of 23%.

### 2.3. Coating Thickness Measurement

For the thickness measurements, coatings were deposited on polished silicon substrates with a size of 20 × 20 mm using the parameters described in Section 2.2. The coating thickness was calculated using a Hommel Tester T1000 profilometer (Jenoptic, Jena, Germany) from not less than 10 measurements. The length of the measured path was 7.5 mm, and the cantilever speed was 0.5 mm/s.

### 2.4. Scaffold Characterization

#### 2.4.1. Scanning Electron Microscopy

The scaffold morphology was studied using scanning electron microscopy (SEM) on a VEGA 3 SBH (Tescan, Brno, Czech Republic) microscope. Before the analysis, the samples were pre-coated with a thin layer of gold using a SmartCoater sputtering system (Jeol, Akishima, Tokyo, Japan). The microscopy was performed in a low vacuum using a secondary electron detector. The diameter of the scaffold fibers was calculated from not less than 100 measurements using the ImageJ 1.38 software (National Institutes of Health, Bethesda, MD, USA).

#### 2.4.2. Atomic Force Microscopy

The high-resolution topography of the scaffold fibers was studied using atomic force microscopy (AFM). The study was performed in semi-contact mode using an NTEGRA Prima (NT-MDT, Moscow, Russia) microscope equipped with a NSG10 (NT-MDT, Russia) cantilever with a 11.8 N/m force constant and a 240 kHz resonance frequency. The AFM images were processed using the Gwyddion 2.63 software (Czech Institute of Metrology, Brno, Czech Republic). The average root mean square roughness (R_ms_) was calculated from not less than five images.

#### 2.4.3. Porosity Measurement

The scaffold porosity was measured using the hydrostatic weighting method described in [17]. The samples with a size of 10 × 10 mm were prepared, and isopropyl alcohol (reagent-grade, Ekos-1, Moscow, Russia) was used as a liquid. The measurement was performed using an AF-224RCE analytical balance (Shinko Denshi, Itabashi-ku, Tokyo, Japan) with VIBRA AFDK (Shinko Denshi, Japan) hydrostatic weighing equipment. The testing was performed five times for each group.

#### 2.4.4. Tensile Testing

The strength and elongation of the scaffolds under uniaxial elongation were studied using an Instron 3344 testing machine (Instron, Norwood, MA, USA) in accordance with ISO 9073-3:1989 standard [18]. The samples were prepared using a manual cutting press (ZCP-020, ZwickRoell GmbH Co. KG, Ulm, Germany) and met the requirements of the ISO 37:2017 standard [19]. The testing was performed five times for each group.

#### 2.4.5. X-Ray Photoelectron Spectroscopy

The elemental composition of the scaffolds was investigated using X-ray photoelectron spectroscopy (XPS) on an XPS NEXSA (Thermo Fisher Scientific, Waltham, MA, USA) spectrometer equipped with a 1486.6 eV Al K Alpha source. The survey spectra were recorded with a maximum energy of 200 eV and a resolution of 1 eV. The analyzed area was a 400 µm^2^ square. The content of chemical elements was calculated from the area of the respective peaks on the survey spectra with a Shirley background subtraction using CasaXPS software (version 2.3.26, Casa Software Ltd., Teignmouth, UK). Three survey spectra were recorded for each sample.

#### 2.4.6. Adhesion of Human Multipotent Mesenchymal Stem Cells

Human multipotent mesenchymal stem cells (MMSCs) were isolated from the subcutaneous adipose tissue of healthy donors. The study was performed in accordance with the Declaration of Helsinki, and approval was obtained from the local Ethics Committee of the Almazov National Medical Research Centre (No. 12.26/2014; 1 December 2014). All the subjects were informed and signed an informed consent prior to a fat tissue biopsy.

The study was performed as described previously [20]. Briefly, after 72 h of cultivation of MMSCs with the tested samples, adhered cells were stained with rhodamine-conjugated phalloidin (Thermo Fisher Scientific, Waltham, MA, USA) and 4,6-diamidino-2-phenylindole (DAPI, dilution 1:40,000, Sigma-Aldrich, USA), followed by fluorescent microscopy Axiovert (Zeiss, Oberkochen, Germany). The non-coated PCL samples and glass slides were used as control groups.

Before the experiment, the samples were sterilized with ethylene oxide in an AN4000 sterilizer (Andersen Sterilizers Inc., Haw River, NC, USA) in accordance with the ISO 10993-7:2008 standard [21] “Biological evaluation of medical devices—Part 7: Ethylene oxide sterilization residuals” standard.

#### 2.4.7. Statistics

The statistical analysis of the obtained results was performed using the Prism 9 software (version 10.4.1, GraphPad Software, Boston, MA, USA) and the Kruskal–Wallis test for the fiber diameter, roughness, tensile strength, elongation, and elemental composition, while the Mann–Whitney test was used for the number of adhered cells and the cell area.

## 3. Results

### 3.1. Scaffold Morphology

It is well known that the average fiber diameter and porosity are the key parameters determining the cell adhesion and proliferation on electrospun scaffolds [22]. The fibers with a diameter of 100–500 nm promote cell proliferation on the scaffold surface but limit the cell migration into the scaffold bulk by forming too small pores. With that, the decrease in the fiber diameter leads to an increase in scaffold rigidity. Fibers with a micrometer diameter and porosity of around 80–90% are preferable, as they support cell adhesion and orientation along the fiber axis and provide the porosity needed for the transport of nutrients and waste products [23]. The scaffolds formed by such fibers allow the migration and proliferation of human dermal fibroblasts and endothelial cells into the scaffold bulk [22], stimulate the formation of new bone tissue in vivo [24], and elicit macrophage anti-inflammatory responses [25].

**Figure 1:**
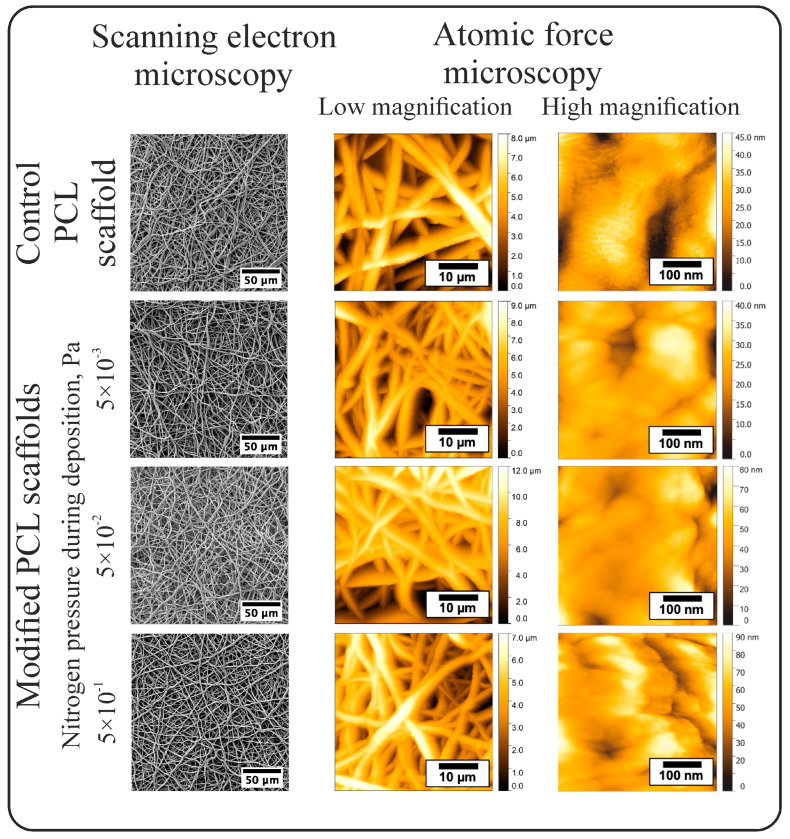
SEM images of the scaffolds and AFM images of the fiber surfaces.

**Figure 2:**
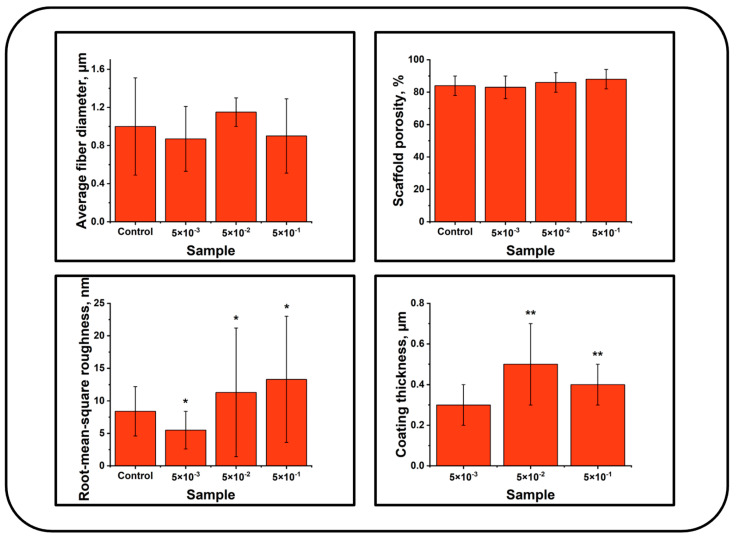
Average fiber diameter, root mean square roughness, coating thickness, tensile strength, and elongation of the scaffolds with N-DLC coatings deposited under various nitrogen pressures. *—*p* < 0.05, statistically significant compared to the control (Kruskal–Wallis test); **—*p* < 0.05, statistically significant compared to the group coated under nitrogen pressure of 5 × 10^−3^ Pa (Kruskal–Wallis test).

The SEM images of the scaffolds and high-resolution images (AFM) of the fiber surfaces coated under various nitrogen pressures are presented in Figure 1. The performed studies demonstrated that the open porous structure (porosity 84 ± 6%) of the electrospun PCL scaffold of the control group (Figure 1) was formed by chaotically interwoven cylindrical fibers with a diameter of 1.11 ± 0.47 µm. Irrespective of the nitrogen pressure, the deposition of N-DLC coatings on the surface of PCL electrospun scaffolds had no effect on the average fiber diameter (Table 1) and did not result in the destruction of the scaffold structure, as evidenced by the absence of fiber breakages, fusions, burns, and other defects on the scaffold surface (Figure 1). Thus, the deposition of N-DLC coatings on the surface of electrospun PCL scaffolds using the PVAD method does not affect the macrostructure of the scaffold (fiber diameter and porosity), preserving the conditions for effective cell adhesion and the transport of nutrients and waste (Figure 1 and Figure 2).

The AFM images of the scaffolds with a low magnification confirm their fibrous structure and the absence of defects on their surface (Figure 1). In the AFM images of the surface of the nonmodified PCL fibers, numerous pores of irregular shape with an area of up to 0.08 µm^2^ were observed (Figure 1). The formation of such pores is typical for electrospun PCL fibers due to the rapid removal of the solvent during the fabrication process [26,27,28]. The root mean square roughness of the fiber surface was found at 8.4 ± 3.8 nm (Figure 2).

Using atomic force microscopy, it was demonstrated that the nitrogen pressure during the PVAD process significantly affects the topography of the fiber surface (Figure 1). On the surface of the fibers coated under a nitrogen pressure of 5 × 10^−3^ Pa, round and ellipsoid clusters with an area of around 0.03 µm^2^ were observed. The clusters had clear boundaries and formed a thin film on the surface and pores of the PCL fibers, resulting in a ~30% decrease in roughness compared to the control fibers (Figure 2). The N-DLC coating deposited under 5 × 10^−2^ Pa fully coated the pores on the fiber surface; the cluster boundaries became unclear, and a “hill”-like topography was observed. These changes resulted in a ~1.9-fold increase in roughness compared to the coatings deposited under a nitrogen pressure of 5 × 10^−3^ Pa (Figure 1 and Figure 2). With an increase in the nitrogen pressure during the deposition process up to 5 × 10^−1^ Pa, the most prominent change in topography was observed. Extended “ridge”-shaped structures up to 85 nm in height and 0.5 µm in length, separated by narrow and deep “canyons”, were found on the surface of the coated fibers. The root mean square roughness of this group was the highest and was found at 13.3 ± 9.7 nm (Figure 2). An increase in the N-DLC coating roughness with an increase in the nitrogen pressure during the PVAD process is typical for such coatings, as observed by Kolpakov et al. [29].

Thus, it was demonstrated that the nitrogen pressure in the chamber is an important technological parameter of N-DLC coating fabrication using the pulsed vacuum arc deposition technique, which allows controlling the topography of the electrospun PCL-based fibers while preserving their structural integrity.

### 3.2. X-Ray Photoelectron Spectroscopy

**Figure 3:**
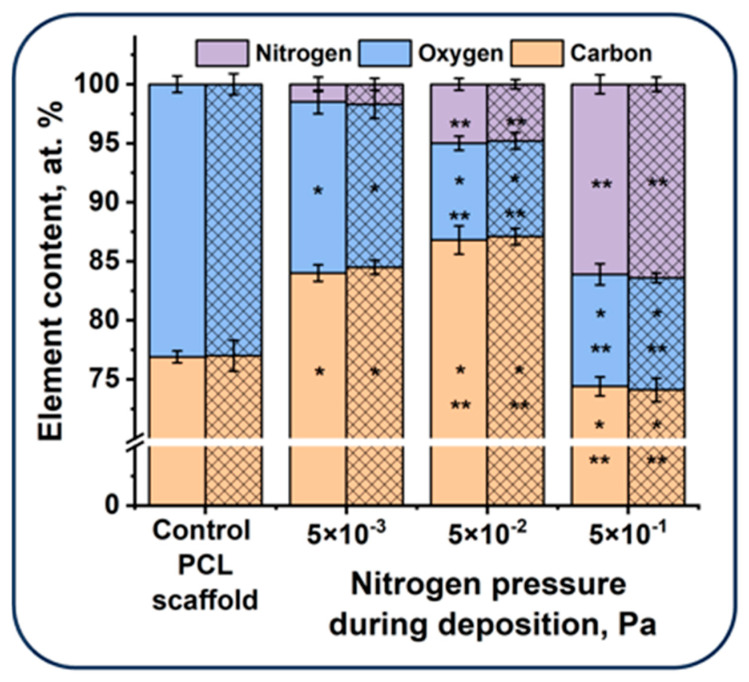
Elemental composition of the scaffold surfaces immediately after the deposition of N-DLC coatings (undashed bars) and after 6 months of storage (dashed bars). *—*p* < 0.05, statistically significant compared to the control (Kruskal–Wallis test); **—*p* < 0.05, statistically significant compared to the group coated under nitrogen pressure of 5 × 10^−3^ Pa (Kruskal–Wallis test).

The elemental analysis of the fabricated scaffolds performed using X-ray photoelectron spectroscopy confirms the incorporation of nitrogen into coatings deposited using PVAD (Figure 3). In the elemental composition of the control electrospun PCL scaffold, carbon and oxygen (76.9 ± 0.5 and 23.1 ± 0.7 at. %, respectively) were found, while nitrogen was not detected. The deposition of a N-DLC coating under a nitrogen pressure of 5 × 10^−3^ Pa resulted in a decrease in the oxygen content to 14.5 ± 1.0 at. % and an increase in the carbon and nitrogen (the elements of the coating) content to 84.0 ± 0.7 and 1.5 ± 0.6 at. %, respectively. With an increase in the nitrogen pressure to 5 × 10^−2^ Pa, the nitrogen content in the N-DLC coating increased to 5.0 ± 0.5 at. %, the oxygen content reduced to 8.2 ± 0.6 at. %, while the carbon content remained unchanged compared to the coating deposited under the nitrogen pressure of 5 × 10^−3^ Pa.

A further increase in the nitrogen pressure resulted in a significant increase in the nitrogen content in the coating, up to 16.1 ± 0.8 at. %. The oxygen content remained the same, and the carbon content returned to the values observed for the control PCL sample. An increase in the nitrogen and sp^2^-carbon content in the N-DLC coatings with an increase in nitrogen pressure during the deposition process were described previously [30].

The XPS of the scaffolds after 6 months of storage did not demonstrate significant changes in the elemental composition of their surface, which leads to the conclusion that a stable disordered C-N alloy was formed under a high nitrogen pressure during the deposition process [30].

Thus, the studies performed demonstrate the possibility of controlling the elemental composition of N-DLC coatings formed on the surface of PCL scaffolds using the PVAD technique. With that, the N-DLC coatings deposited on the surface of the electrospun PCL scaffolds preserve their elemental composition after storage for 6 months under normal conditions.

### 3.3. Tensile Testing

**Figure A1:**
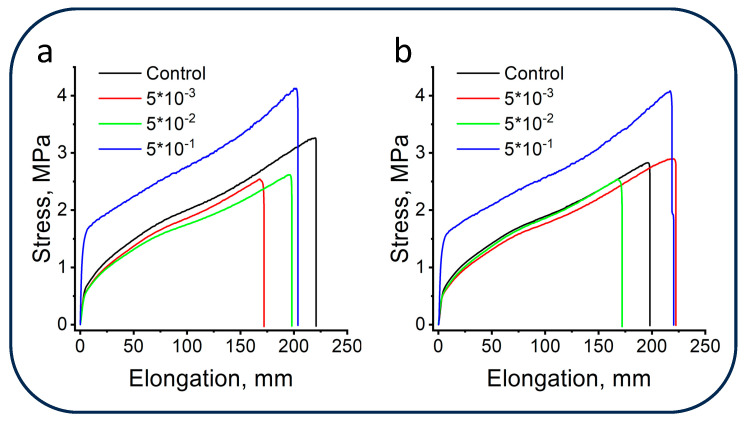
Typical stress–strain curves of the uncoated and coated scaffolds immediately after the coating deposition (**a**) and after 6 months of storage (**b**).

The results of the tensile testing of the electrospun PCL scaffolds with N-DLC coatings deposited under various nitrogen pressures are presented in Table 1 and Appendix A, Figure A1. The scaffolds of the control group demonstrated a 2.9 ± 0.3 MPa tensile strength and a high elongation of 428 ± 42%, which is typical of the PCL scaffolds fabricated using the electrospinning technique [2]. Irrespective of the nitrogen pressure, the deposition of N-DLC coatings had no effect on the scaffold’s elongation, while the tensile strength changed significantly (Table 1). The formation of an N-DLC coating under a nitrogen pressure of 5 × 10^–3^ resulted in a ~20% decrease in the tensile strength compared to the control PLC scaffold. With an increase in the nitrogen pressure up to 5 × 10^−2^, the tensile strength increased to the values of the control scaffold (Table 1). The maximum tensile strength was more than 40% higher compared to the control scaffold for the scaffolds with an N-DLC coating deposited under a 5 × 10^−1^ Pa nitrogen pressure.

The influence of the nitrogen pressure during the deposition process on the tensile strength of the coated scaffolds may be explained as follows. On the one hand, the high energy of the carbon ions during the deposition of the N-DLC coatings using the PVAD technique explains the high adhesion of the coating to the substrate [31]. On the other hand, the doping of DLC coatings with nitrogen is known as an approach for controlling the internal stress in the coating. An increase in the nitrogen content results in the formation of films with a lower internal stress [30]. These facts lead us to the suggestion that the coatings deposited under a nitrogen pressure of 5 × 10^−3^ possess the highest internal stress, resulting in the formation of defects on the substrate surface and the reduction in the scaffold tensile strength. It was also observed that an increase in the nitrogen pressure during the deposition process resulted in more than a 1.5-fold increase in the coating thickness (from 0.3 ± 0.1 to 0.5 ± 0.2 µm, Figure 2), which is in agreement with the results of Koskinen et al. [32]. Thus, an increase in the nitrogen pressure increases the coating thickness and nitrogen content in the coating and decreases the internal stress, reducing the destructive effects on the fiber surface. On the other hand, because of its high strength, the N-DLC coating reinforces the scaffold. Furthermore, the increased nitrogen content increases the adhesion of the N-DLC coating to the substrate through the formation of a “carbon-nitrogen” bond [33].

The tensile testing of the PCL-based scaffolds after 6 months of storage revealed no statistically significant changes (Table 1). That effect is due to the high stability of the N-DLC coatings [34] and a low degradation rate of PCL compared to the other biodegradable polyesters (e.g., polylactic acid, polyglycolic acid, etc.) [35] under normal conditions.

Thus, the performed studies demonstrated that the nitrogen pressure in the chamber during the deposition of the N-DLC coatings using the PVAD technique on the surface of electrospun PCL scaffolds is an efficient tool for the control of the mechanical characteristics of the resulting materials. It should be highlighted that the strength and elongation of the fabricated scaffolds are close to the mechanical characteristics of the femoral and internal mammary arteries, subcutaneous veins, heart valves, some muscles, human periosteum, and stomach [36]. With that, the tensile properties of the modified scaffolds were preserved after 6 months of storage.

### 3.4. MMSC Adhesion

**Figure 4:**
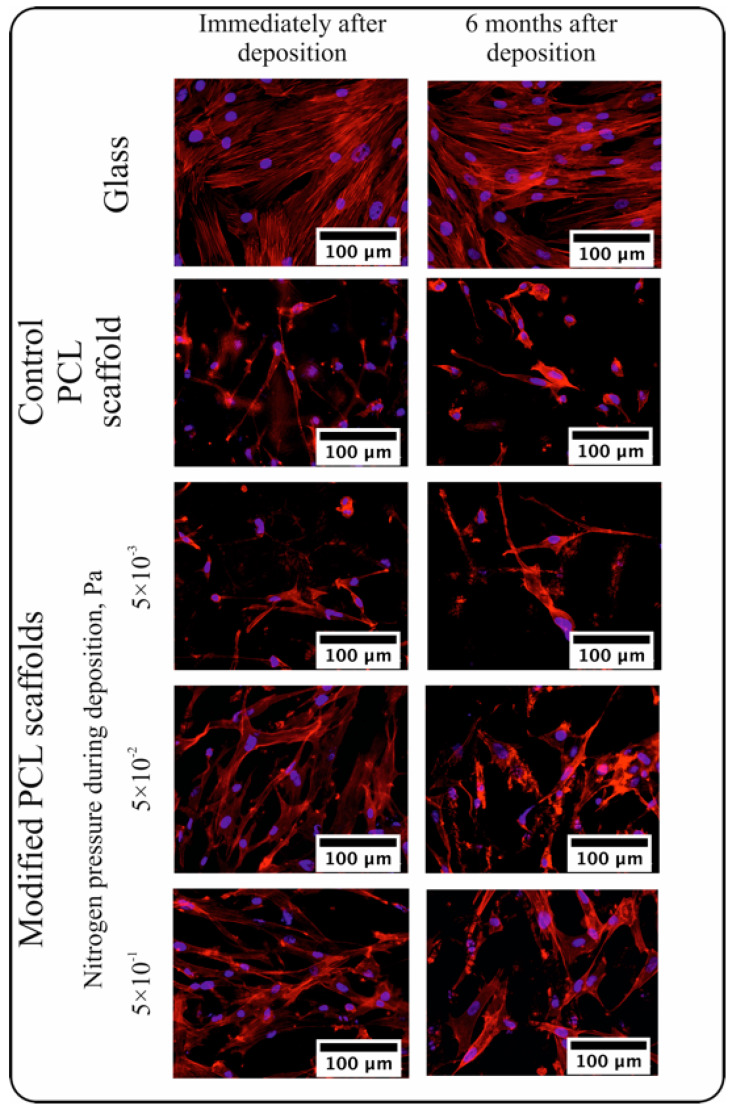
MMSCs adhered to the surface of the fabricated scaffolds (×40 magnification). (**Left**) column—the cells cultured on the samples immediately after N-DLC coating deposition, (**right**) column—the cells cultured on the samples stored for 6 months. Cells cytoplasm stained in red, cells nuclei stained in purple blue.

The fluorescent microscopy images of human MMSCs cultured on the surface of scaffolds with the N-DLC coatings deposited under various nitrogen pressures immediately after the deposition and after 6 months of storage are presented in Figure 4.

**Figure 5:**
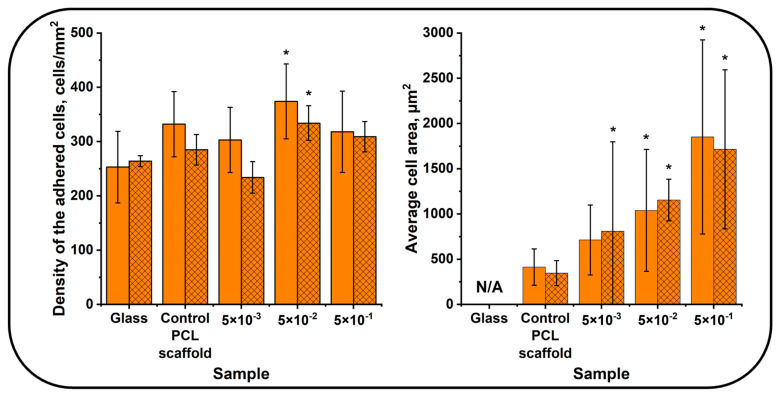
The density and morphology of MMSCs adhered to the surface of electrospun PCL scaffolds with N-DLC coatings deposited under various nitrogen pressures immediately after the deposition of N-DLC coatings (undashed bars) and after 6 months of storage (dashed bars). *—*p* < 0.05, statistically significant compared to control (Mann–Whitney test).

The used human MMSC culture was viable, as confirmed by the formation of a confluent layer after 72 h of cultivation on glass in both experiments. The density of the adhered cells was 332 ± 60 cells/mm^2^ in the experiment with the PCL scaffold immediately after the N-DLC coating deposition and 285 ± 28 cells/mm^2^ after 6 months of storage (Figure 4 and Figure 5). The cells had an area of 413 ± 201 µm^2^ and adhered mainly to the surface. The cells had a typical elongated shape with a small number of pseudopodia connected to the other cells (Figure 4). Individual cells had a round shape with pseudopodia.

The number of cells adhered to the surface of the PCL scaffold was found to be higher compared to the number of those adhered to glass. This may be explained by the larger surface available for cell adhesion. Despite the higher number of adhered cells, their round shape, low area, and number of pseudopodia demonstrate the non-optimal conditions for adhesion and proliferation of cells on the surface of the pristine PCL scaffolds [37].

The number and area of cells adhered to the surface of the electrospun PCL scaffolds with an N-DLC coating deposited under a nitrogen pressure of 5 × 10^−3^ Pa did not change significantly compared to the control PCL scaffold. The cells had a low number of pseudopodia for intercellular interactions. It may be concluded that the N-DLC coating deposited under these conditions did not enhance the biocompatibility of the electrospun PCL scaffold (Figure 5).

The increased number and area of the adhered cells were observed after the cultivation with the samples coated under a nitrogen pressure of 5 × 10^−2^ Pa (Figure 4 and Figure 5). The cells were oriented along the fibers, had a high area and numerous pseudopodia, and interacted with the other cells and sample surface. Actin microfilaments were clearly visualized, and both proliferating and rounded cells were observed on the sample surface.

The area of the cells cultured on the samples coated under the nitrogen pressure of 5 × 10^−1^ Pa was found to be the highest observed during the experiments (Figure 5). The adhered cells had the highest area with clearly visualized actin microfilaments. Only a few round cells were observed.

The investigation of the adhesion process on the scaffolds stored for 6 months demonstrated that it was not worse than on the as-received samples (Figure 4 and Figure 5).

The observed results demonstrate that the formation of an N-DLC coating using the PVAD technique in a nitrogen atmosphere is an efficient approach for the enhancement of the biocompatibility of electrospun PCL-based scaffolds. The adjustment of the nitrogen pressure during the deposition process provides various conditions for the adhesion and proliferation of human MMSCs on the surface of PCL-based scaffolds, and an increase in the nitrogen pressure leads to the enhanced biocompatibility. It should be noted that scaffolds with N-DLC coatings are able to preserve high biocompatibility even after 6 months of storage under normal conditions.

The observed effect may be explained as follows. Microtopography and chemical composition are known to be the key factors that affect cell adhesion and proliferation [37]. It was demonstrated that a higher nitrogen pressure results in the deposition of coatings with an increased nitrogen content (Figure 2). It is well known that N-DLC coatings enhance the cell adhesion to glass surfaces, presumably due to the hydrophilicity optimal for protein absorption [38]. An increase in the nitrogen content in the N-DLC coatings deposited on the surface of the polished silicon slides also resulted in an enhanced cell adhesion [39]. On the other hand, the increased roughness was observed with an increase in the nitrogen pressure during coating deposition (Figure 1, Table 1). Nanotopography significantly affects cell adhesion and proliferation, and the surfaces with the optimal roughness ratio stimulate cell adhesion [40,41]. In our work, the increase in the N-DLC surface roughness was observed with the increase in the nitrogen pressure during the deposition process. The observed enhancement of MMSC adhesion correlated with the nitrogen concentration in the deposited coatings and with the coatings’ roughness. Thus, the formation of thin N-DLC coatings on the surface of electrospun PCL scaffolds solves two problems simultaneously: the creation of the optimal elemental composition and nanotopography on the surface of the electrospun PCL scaffold. With that, the high ability of nitrogen for DLC ligation and the formation of a stable disordered C–N alloy determine the high ability of N-DLC coatings deposited using the PVAD technique to preserve their elemental composition, providing a high biocompatibility even after long-term storage.

## 4. Conclusions

The performed studies demonstrate pulsed vacuum arc deposition as a promising technique for the fabrication of nitrogen-doped diamond-like carbon coatings on the surface of electrospun poly(ε-caprolactone) scaffolds. The variation in the nitrogen pressure during the deposition process is an efficient tool for controlling the elemental composition, roughness, and thickness of the coatings, as well as the mechanical and biological characteristics of the modified scaffolds. Particularly, the coatings deposited under a nitrogen pressure in the range of 5 × 10^−2^ to 5 × 10^−1^ Pa provided the best conditions for the adhesion of human mesenchymal stem cells. The main advantage of the suggested modification technique is that it allows for the deposition of nitrogen-doped diamond-like carbon coatings onto the surface of electrospun poly(ε-caprolactone) microfibers with a low melting point. This enhances the cell adhesion and proliferation without causing any damage to the scaffold structure. The main feature of the deposited nitrogen-doped diamond-like carbon coatings is the preservation of their elemental composition, tensile properties, and enhanced cell adhesion to their surface even after 6 months of storage under standard conditions. This opens new opportunities for the manufacturing of these materials and their application in tissue engineering.

## Figures and Tables

**Table 1:** Tensile strength and elongation of the scaffolds with N-DLC coatings deposited under various nitrogen pressures.

Nitrogen Pressure, Pa	Tensile Strength, MPa		Elongation, %	
	Immediately After Deposition	After 6 Months of Scaffold Storage	Immediately After Deposition	After 6 Months of Scaffold Storage
Control PCL scaffold	2.9 ± 0.3	3.0 ± 0.2	430 ± 40	390 ± 60
5 × 10^−3^	2.3 ± 0.1 *	2.5 ± 0.2 *	400 ± 20	420 ± 30
5 × 10^−2^	3.0 ± 0.4	2.9 ± 0.3	400 ± 40	380 ± 40
5 × 10^−1^	4.2 ± 0.7 *	3.9 ± 0.5 *	400 ± 100	390 ± 80

*—*p* < 0.05, statistically significant compared to the control (Kruskal–Wallis test).

## Data Availability

The raw data supporting the conclusions of this article will be made available by the authors on request.
